# The impact of force magnitude on the first and second maxillary molars in cervical headgear therapy

**DOI:** 10.1093/ejo/cjab010

**Published:** 2021-04-03

**Authors:** Tuula Talvitie, Mika Helminen, Susanna Karsila, Reeta Varho, Luca Signorelli, Pertti Pirttiniemi, Timo Peltomäki

**Affiliations:** Oral Diseases, The Hospital District of South Ostrobothnia, Seinäjoki, Finland; Institute of Dentistry, Faculty of Health Sciences, University of Eastern Finland, Kuopio, Finland; Tays Research Services, Tampere University Hospital, Tampere, Finland; Faculty of Social Sciences, Health Sciences, Tampere University, Tampere, Finland; Dental Teaching Unit, Turku Municipal Health Care Services, Turku, Finland; Dental Teaching Unit, Turku Municipal Health Care Services, Turku, Finland; Private practice, Wil, Switzerland; Department of Oral Development and Orthodontics, Research Unit of Oral Health Sciences, University of Oulu, Finland and Medical Research Center, Oulu University Hospital, Oulu, Finland; Institute of Dentistry, Faculty of Health Sciences, University of Eastern Finland, Kuopio, Finland; Faculty of Medicine and Health Technology, Tampere University, Tampere, Finland; Department of Oral and Maxillofacial Diseases, Kuopio University Hospital, Kuopio, Finland; Department of Ear and Oral Diseases, Tampere University Hospital, Tampere, Finland

## Abstract

**Aim:**

To study the effect of force magnitude on the maxillary first and second molars in cervical headgear (CHG) therapy.

**Material and methods:**

In this controlled clinical trial, patients (*n* = 40) were treated with CHG with a light (L, 300 g, *n* = 22) or a heavy force (H, 500 g, *n* = 18) magnitude. The subjects were asked to wear CHG for 10 hours a day for 10 months. The outer bow of the CHG facebow was lifted up for 10–20 degrees and the inner bow was expanded 3–4 mm. Adherence to instructions and force magnitude were monitored using an electronic module (Smartgear, Swissorthodontics, Switzerland). Panoramic and lateral radiographs before (T1) and after treatment (T2) were analysed using a Romexis Cephalometric module (Planmeca, Finland) focussing on the angular, sagittal, and vertical positions of the permanent first and second molars.

**Results:**

According to the cephalometric analysis of the maxillary first and second molars, distal tipping occurred during T1–T2 in the H group (*P* = 0.010 and 0.000, respectively), and the change was greater in the H group compared to the L group (*P* = 0.045 and 0.019, respectively). Based on the panoramic analysis, tipping occurred in the distal direction during therapy in the H group in the second molars compared to the midline or condylar line (*P* = 0.001 and 0.001; *P* = 0.008 and 0.003 on the right and left, respectively).

**Conclusion:**

With heavy force magnitude, the maxillary first and second molars can tilt more easily in the distal direction even if the CHG was used less. Distal tipping of the molar can be considered to be a side effect of CHG therapy.

## Introduction

A common objective in Class II therapy with headgear (HG) is the distal movement of the maxillary first permanent molars to a Class I molar relationship and gain space on a crowded dental arch by lengthening the upper dental arch (1,2). However, with distal movement, the maxillary first molars often tilt in a posterior direction (2–8) and the upper second molars appear to move distally with the first molars (4, 6, 7). The unerupted maxillary second molars allow the first molars larger movement and tipping in the distal direction (7, 9). Tipping of the first molars during HG treatment can evidently be controlled by different kinds of activation of the extra-oral bow. It has been suggested that a larger component of cervical directed force in the HG results in greater tipping of the maxillary first molars (3, 10, 11). By bending the long outer extra-oral bows upwards in the cervical headgear (CHG), the line of force moves more cranially and prevents distal tilting of the upper molars (11–14).

It has been reported that the use of heavy force magnitude in CHG therapy causes easier extrusion of the upper first molars and, as a consequence, posterior rotation of the mandible (7, 10, 15). However, several studies have reported no posterior rotation of the lower jaw related to CHG treatment (1, 4, 16). On the contrary, anterior rotation of the lower jaw during and after CHG therapy has been reported (17).

When the maxillary first molars are moved and tipped in the distal direction, the eruption path of the second molars can be blocked, causing delayed eruption (6, 18, 19). It is not only the activation of the HG but also the development and eruption stage of the maxillary second molars that are thought to influence the effectiveness of maxillary first molar movement in the distal direction and the change in angulation in HG therapy (7, 20–22). It is important to identify the factors that influence tipping and distalization of the maxillary first and second molars during HG treatment.

The use of CHG differs greatly depending on how an orthodontist wants to operate with the appliance. The guidelines suggest various combinations of activation (23). Initiation of the treatment varies, from 7 to 17 years (4, 11, 24–26); therapy can last for a short period or for several years, from 6 months up to 6 years (2). The applied force in CHG can vary from light to very heavy, 200 to 1000 g (1, 8, 15, 25, 27–29). Activation of the face bow can vary significantly; the inner arch can be activated with or without expansion and the long outer bow can be used at the same level as the inner bow or can be bent upwards or downwards (5, 8, 11, 26, 30). Additionally, recommended wear time varies from 8 hours of night-time use to 16 hours or even full-time use (1, 8, 11, 26, 31).

A force magnitude in HG therapy over 450 g is assumed to surpass the threshold potential of a tooth movement and the effect has, therefore, been considered to be more orthopaedic (skeletal); less than 450 g is considered to primarily cause dental effects (12, 13). Based on our previous study, no pure dental or skeletal effects have been achieved in CHG therapy even though this threshold value has been applied (16, 32). It has been found that force magnitude is not stable during CHG use (16, 32); head posture and direction of traction are thought to have an effect on the amount of active force, and force fluctuation constantly occurs during use, indicating that CHG is really being actively used (32–35).

Despite the widespread use and study of HG, it is still unclear how the differences in force magnitude influence the maxillary first and second molars. The aim of this controlled clinical trial was to evaluate the effect of the force magnitude of CHG on the angular, vertical, or sagittal position of the maxillary first and second molars when the maxillary second molars had not yet erupted.

## Subjects and methods

Subjects and methods have been described in previous publications (16, 32). However, for the sake of clarification, they are briefly included here. The subjects in the present study were recruited from a pool of children eligible for treatment at the Health Care Center of Turku in Finland. The subjects were deemed to be potential research subjects during screening if they met the following clinical criteria:

1. Class II (or end-to-end) molar relationship
2. Mixed dentition stage
3. Moderate dental crowding

A thorough orthodontic examination was performed, including radiological examination (panoramic and lateral radiograph), dental and facial photography, as well as impressions for study models. The treatment plan was created by an experienced orthodontist (SK). If the initial treatment method was solely CHG, the subject was invited to participate. Patients and parents gave their informed consent. The Ethical Committee of the Hospital District of Southwest Finland approved the research plan (ETMK: 77/180/2011).

Since the patients were to be treated as part of clinical orthodontic education at the Institute of Dentistry, University of Turku, the goal of the recruitment was a minimum of 44 patients (one CHG patient for each student). A pre-study power analysis was also conducted to determine an appropriate sample size for the study. Based on the findings of a previous study, it was estimated that a 3-mm difference in upper dental arch length gain over 12 months of CHG use could be expected (1). It was further assumed that the standard deviation (SD) would be the same, that is, 3 mm, in the light and heavy force magnitude groups. To achieve 80 per cent statistical power with a 5 per cent significance criterion, 17 subjects would be needed in each group.

Once informed consent had been obtained, a receptionist made an appointment to make the CHG. The CHG was made by a dental student, although all patients were closely supervised during all phases by an experienced orthodontist (SK) and a postgraduate in orthodontist (RV). The inner bow of the CHG was expanded (3–4 mm) and the long outer bow bent 10–20 degrees upwards in relation to the inner bow. The patients were allocated into two groups: a light (L) and heavy (H) group, and the force magnitude was set to 300 and 500 g, respectively. The force magnitude was set while the patient was sitting and looking straight ahead. First, 22 patients from the random recruitment list were given CHG with a light force because, at the time the students’ course started, only light force modules were available.

**Figure 1.**
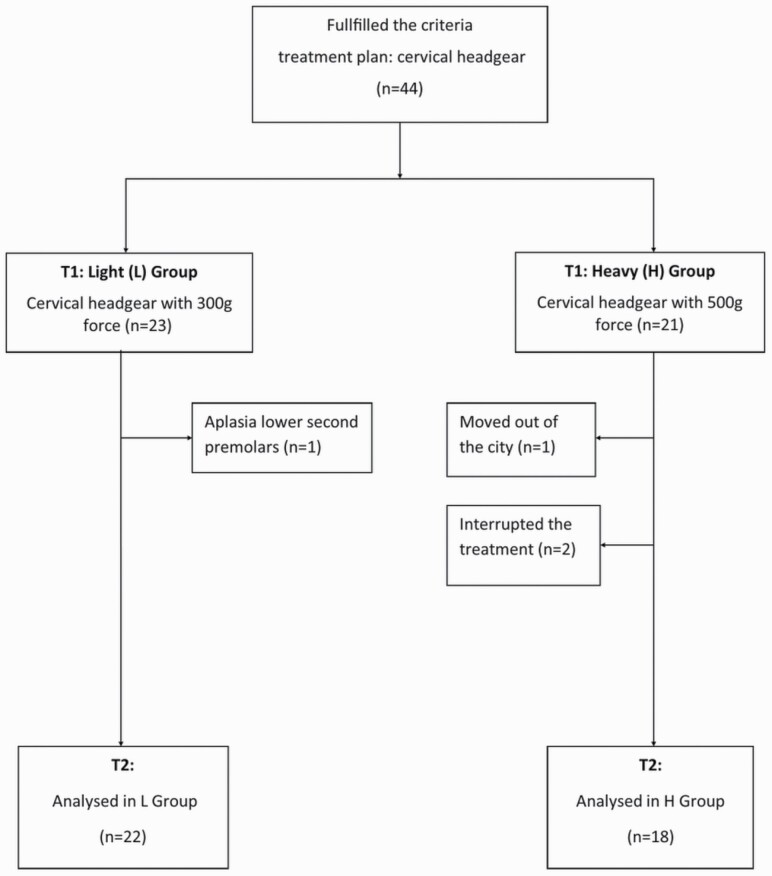
Chart illustrating the flow of subjects T1–T2.

The patients were advised to wear the CHG for 10 hours, that is, when they were sleeping. However, the importance of wearing the CHG in the early evening hours was also emphasized. The patients were seen every 6–8 weeks until the end of the study period after 10 months, and the force and use of the CHG was controlled and readjusted on these occasions. Adherence to instructions and force magnitude in CHG use were monitored via an electronic module (Smartgear, Swissorthodontics, Switzerland) integrated into the HG’s neck strap on the right side. For safety reasons, a snap-away spring mechanism was used on both sides. During the study, both children and their parents did not know which group—L or H—they belonged to, although they knew that they were participating in a clinical trial. Initially, 44 children were recruited but, during the study, two left, one moved away from the city, and one dropped out due to aplasia in the lower permanent premolars, which had been noted after the treatment. Thus, the present study comprises 40 children: 22 in the L group (8 male and 14 female) and 18 in the H group (7 male and 11 female). Patient flow is described in the chart flow (Figure 1). The first 6–8 weeks were an adjustment period using a 300-g force in both groups. During this period, the patients were instructed to gradually learn to fit the CHG and sleep while wearing it.

**Figure 2.**
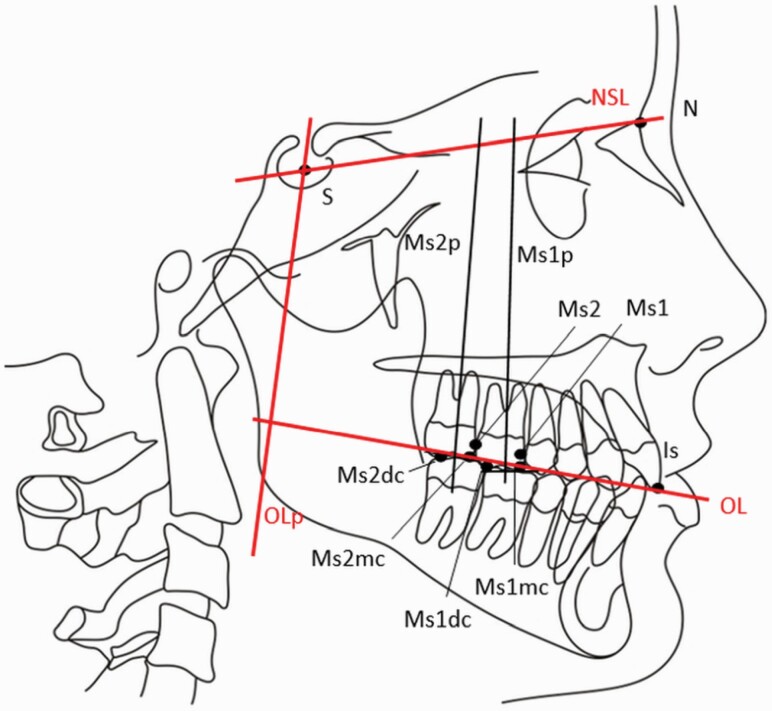
Landmarks and planes used in the cephalometric analysis. S: sella, used as a reference point for all lateral cephalograms. N: nasion, the most anterior point of the frontonasal suture. Is: incisal tip of the maxillary incisor; the incisal tip of the most anterior maxillary central incisor. Ms1mc: maxillary first molar, mesial cusp. The mesiobuccal cusp tip of the maxillary permanent first molar; the midpoint was used if a double projection was seen. Ms1dc: maxillary first molar, distal cusp. The distobuccal cusp tip of the maxillary permanent first molar; the midpoint was used if a double projection was seen. Ms2mc: maxillary second molar, mesial cusp. The mesiobuccal cusp tip of the maxillary permanent second molar; the midpoint was used if a double projection was seen. Ms2dc: maxillary second molar, distal cusp. The distobuccal cusp tip of the maxillary permanent second molar; the midpoint was used if a double projection was seen. NSL: sella–nasion line, plane S–N. Used as a linking factor for all lateral cephalograms. OL: occlusal line (T1), plane Ms1mc-Is. Used as a reference plane and copied from the original (T1) to T2 lateral cephalogram. OLp: occlusal line perpendicular; the plane perpendicular to OL through *S*. Used as a reference plane and copied from the T1 to T2 lateral cephalogram. Ms1p: perpendicular line to the occlusal line of the first maxillary molar (Ms1mc–Ms1dc). Ms2p: perpendicular line to the occlusal line of the second maxillary molar (Ms2mc–Ms2dc). MS1: maxillary first molar mesial contact point. MS2: maxillary second molar mesial contact point.

**Figure 3.**
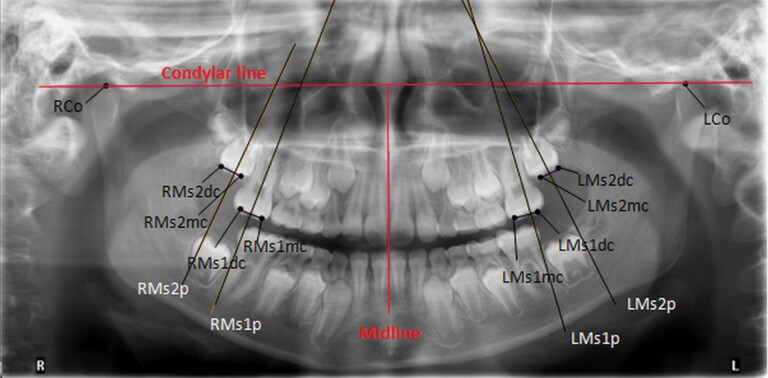
Landmarks used in the panoramic analysis. RMs1p: perpendicular line to the occlusal line of the right first maxillary molar (Ms1mc–Ms1dc). RMs2p: Perpendicular line to the occlusal line of the right second maxillary molar (Ms2mc–Ms2dc). RMs1mc: right first maxillary molar mesial cusp. The mesiobuccal cusp tip of the right maxillary permanent first molar; the midpoint was used if a double projection was seen. RMs1dc: right first molar superius distal cusp. The distobuccal cusp tip of the maxillary permanent first molar; the midpoint was used if a double projection was seen. RMs2mc: right second molar superius mesial cusp. The mesiobuccal cusp tip of the maxillary permanent second molar; the midpoint was used if a double projection was seen. RMs2dc: right second molar superius distal cusp. The distobuccal cusp tip of the maxillary permanent second molar; the midpoint was used if a double projection was seen. RMs1: the occlusal line of the right maxillary first molar. Plane RMs1mc–RMs1dc. Rms2: the occlusal line of the right maxillary second molar. Plane RMs2mc–RMs2dc. RCo: the most superior point of the right condyle. LMs1p: perpendicular line to the occlusal line of the left maxillary first molar (Ms1mc–Ms1dc). LMs2p: perpendicular line to the occlusal line of the left maxillary second molar (Ms2mc–Ms2dc). LMs1mc: maxillary left first mesial cusp. The mesiobuccal cusp tip of the right maxillary permanent first molar; the midpoint was used if a double projection was seen. LMs1dc: left first molar superius distal cusp. The distobuccal cusp tip of the maxillary permanent first molar; the midpoint was used if a double projection was seen. LMs2mc: left second molar superius mesial cusp. The mesiobuccal cusp tip of the maxillary permanent second molar; the midpoint was used if a double projection was seen. LMs2dc: left second molar superius distal cusp. The distobuccal cusp tip of the maxillary permanent second molar; the midpoint was used if a double projection was seen. LMs1: the occlusal line of the left maxillary first molar. Plane LMs1mc–LMs1dc. LMs2: the occlusal line of the left maxillary second molar. Plane LMs2mc–LMs2dc. LCo: the most superior point of the left condyle. Condylar line: reference line RCo–LCo. Midline: line following the midsagittal sutura.

Panoramic and lateral radiographs were taken at the beginning and the end of the study (10 months). The cephalograms were analysed using a modified Pancherz analysis focussed on maxillary molar angulation and position compared to the reference lines. In cases in which two contours were seen, the midpoint of the counters was used for the analysis (36,37) (Figure 2). In the panoramic analysis, the angulation of the maxillary first and second molars was evaluated according to the Hadler–Olsen method (38) (Figure 3). The maxillary second molar development stage was evaluated from the panoramic radiograms, classified into eight groups according to Demirjian’s classification (39) (Table 1). The eruption stage of the second molars was compared to the adjacent first molar and divided into five different groups (40, 41) (Table 2). An analysis was conducted using a Romexis Cephalometric module (Planmeca, Finland) by one person (TT), who was blinded for which group patient was attended to.

**Table 1.** The maxillary second molar development stage was evaluated from the panoramic radiographs, classified into eight groups according to Demirjian’s classification (39)

The development stage of the maxillary second molar at T1	Light force (300 g)		Heavy force (500 g)		Total	
	Right	Left	Right	Left	Right	Left
The beginning of the calcification is seen at the cusp tips	0	0	0	0	0	0
	0%	0%	0%	0%	0%	0%
Fusion of the mineralized tips is seen	0	0	0	0	0	0
	0%	0%	0%	0%	0%	0%
The crown formation is at the halfway point	0	0	0	0	0	0
	0%	0%	0%	0%	0%	0%
The crown is completed to the cementoenamel junction	14	11	6	7	20	18
	63.6 %	50.0%	33.3%	38.9 %	50.0%	45.0%
The root length is less than the crown height; the initial formation of a bifurcation area is seen	4	8	7	6	11	14
	18.2%	36.4%	38.9%	33.3%	27.5%	35.0%
The root length is at least the crown height; roots have funnel-shaped endings	4	3	5	5	9	8
	18.2%	13.6%	27.8%	27.8%	22.5%	20.0%
The root walls are parallel and the apex is still open	0	0	0	0	0	0
	0%	0%	0%	0%	0%	0%
The apex is closed and tooth formation is completed	0	0	0	0	0	0
	0%	0%	0%	0%	0%	0%
Total	22	22	18	18	40	40
	100%	100%	100%	100%	100%	100%

**Table 2.** The eruption stage of the maxillary permanent second molar was classified according to the adjacent maxillary permanent first molar (40, 41)

Eruption stage of maxillary second molar at T1	Light force (300 g)		Heavy force (500 g)		Total	
	Right	Left	Right	Left	Right	Left
The occlusal surface of the maxillary second molar is at the same level as the occlusal level of the adjacent first molar.	0	0	0	0	0	0
	0%	0%	0%	0%	0%	0%
The occlusal surface of the maxillary second molar is positioned occlusally to the cementoenamel junction of the adjacent first molar.	0	0	0	2	0	2
	0%	0%	0%	11.1%	0%	5.0%
The occlusal surface of the maxillary second molar is at the same level as the cementoenamel junction of the adjacent first molar.	2	4	5	3	7	7
	9.1%	18.2%	27.8%	16.7%	17.5%	17.5%
The occlusal surface of the maxillary second molar is positioned apically to the cementoenamel junction of the adjacent first molar.	20	18	13	13	33	31
	90.9%	81.8%	72.2%	72.2%	82.5%	77.5%
The occlusal surface of the maxillary second molar is positioned cranially to the apex of the adjacent first molar.	0	0	0	0	0	0
	0.0%	0.0%	0.0%	0.0%	0.0%	0.0%
Total	22	22	18	18	40	40
	100%	100%	100%	100%	100%	100%

### Statistical analysis

A Mann–Whitney *U*-test was used when the measurements were evaluated between the groups (L and H) at time points T1, T2, and T1–T2. A Wilcoxon test was used to analyse the changes during the study period (T1–T2) within the group (L or H). The association between the change of position in the maxillary first and second molars (T1–T2) and the development and eruption stages of the maxillary second molars at T1 was evaluated using Spearman’s rank correlation coefficients. *P* values less than 0.05 were considered to be statistically significant.

All linear and angular measurements were made twice at a minimum of 2-week intervals, and the mean value was used for statistical analysis. From the panoramic radiograph, the development and eruption stage of the second molars was evaluated; a more advanced stage (right or left) was used for the correlation analysis. Interclass correlation coefficients (ICCs) was used to evaluate the reliability of the first and second measurements.

## Results

In the cephalometric measurements, ICC values varied from 0.692 to 0.964. The highest value was in the distance Ms2mc–NSL (0.964) and the lowest in the angle NSL/MS2p (0.692). In the panoramic angular measurements, ICC values varied from 0.712 to 0.955. The lowest value was in the angle midline/LMs1p (0.721) at T2 and the highest in the angle condylar line/RMs2p (0.955). A table about ICC and measurement error is available online (Supplementary Material).

At the beginning of the therapy, no age difference between the groups was found. The mean age of the subjects at T1 was 9.7 years (SD 0.73 years) and 9.9 years (SD 0.74 years) in the L and H groups, respectively.

All patients had unerupted maxillary second molars at the start of the study. In 82.5 and 77.5 per cent of the second molars on the right and left side, respectively, the occlusal surface of the second molars was positioned apically to the cementoenamel junction of the adjacent first molars at T1. The most advanced stage of eruption of the maxillary second molars was detected as being at the same level of the cementoenamel junction of the adjacent first molars (Table 1). An assessment of the development stage of the maxillary second molars showed that 50.0 and 45.0 per cent on the right and left side, respectively, had the crown formation completed and, in the most developed cases, root formation had reached the crown height with funnel-shaped endings (Table 2). At the end of the study, most of the children still had unerupted maxillary second molars, with the exception of five (all in the H group), where at least one maxillary second molar had clinically partially erupted. At T1, no statistically significant differences between the groups were found in the cephalometric or the panoramic analysis of linear and angular measurements and in the development or eruption stage.

### Cephalometric analysis

**Figure 4.**
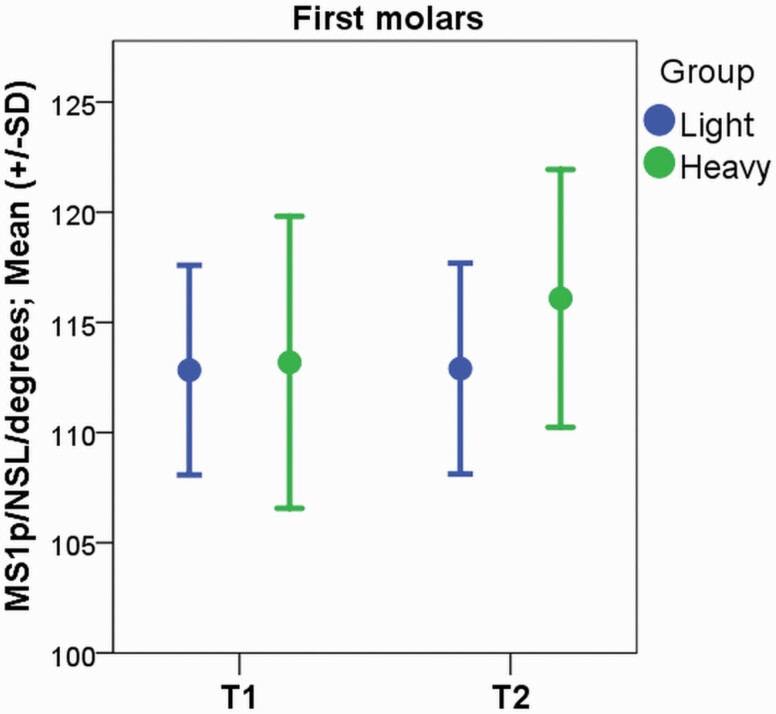
In the cephalometric analysis, the maxillary first molars were found to tip distally with a heavy force during cervical headgear therapy (*P* = 0.010), with great individual variability.

**Figure 5.**
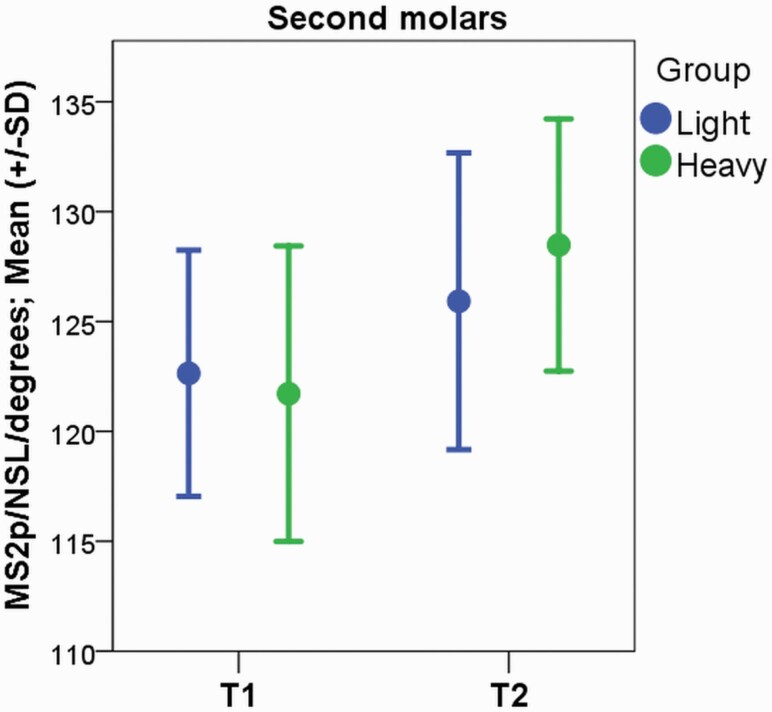
In the cephalometric analysis, the maxillary second molars were found to tip distally with a heavy force during cervical headgear therapy (*P* = 0.000), with great individual variability.

The results of the cephalometric analysis are presented in Table 3. During the treatment, the maxillary first and second molars tipped in the distal direction with great individual variability in the H group (2.90 degrees, SD 4.12 degrees, *P* = 0.000 and 6.77 degrees, SD 5.99 degrees, *P* = 0.000 in the first and second molars, respectively; Figures 4 and 5). In the L group, no change in the angulation of the maxillary first or second molars during therapy was noted. In both groups, the maxillary first and second molars erupted towards the occlusal plane (L: 1.35 mm, SD 1.12, *P* = 0.000; H: 1.50 mm, SD 0.83 mm, *P* = 0.000 and L: 1.64 mm, SD 1.17 mm, *P* = 0.000 and H: 1.90 mm, SD 0.90 mm, *P* = 0.000 in the first and second molars, respectively). The position of the maxillary first molars changed in the distal direction in the L and H groups with great individual variability (−0.78 mm, SD 1.72 mm, *P* = 0.030 and −1.98 mm, SD 1.71 mm, *P* = 0.000, respectively). In the H group, statistically significant movement of the maxillary second molars in the distal direction was noted (−1.87 mm, SD 1.61 mm, *P* = 0.000). At the end of the trial (T2), no differences between the groups were found in the cephalometric analysis, although the change in the angulation of the maxillary first (L: 0.07 degrees, SD 3.91 degrees, H: 2.90 degrees, SD 4.12 degrees, *P* = 0.045) and second molars (L: 3.28 degrees, SD 7.12 degrees, H: 6.77 degrees, SD 5.99 degrees, *P* = 0.019) was larger in the H group, with great individual variability.

**Table 3.** During the treatment, the following statistically significant changes were found: eruption of the maxillary first and second molars in both groups; distal tipping of the maxillary first and second molars in the H group; movement in the distal direction of the maxillary first molars in both groups; movement in the distal direction of the maxillary second molars in the H group. The changes in the angulation of the molars were greater in the H group. Negative values signify movement in the distal direction

Cephalometric analysis		Light force, L (300 g)							Heavy force, H (500 g)							L vs. H	L vs. H	L vs. H
		T1		T2		T1–T2		P*	T1		T2		T1–T2		P*	T1	T2	T1–T2
*N* = 40	Valid	22		22		22		22	18		18		18		18	40	40	40
		Mean	SD	Mean	SD	Mean	SD		Mean	SD	Mean	SD	Mean	SD		*P*	*P*	*P*
NSL/Ms1p (°)		112.83	4.76	112.90	4.79	0.07	3.91	1.000	113.19	6.63	116.09	5.85	2.90	4.12	**0.010**	0.619	0.066	**0.045**
NSL/Ms2p (°)		122.64	5.61	125.92	6.75	3.28	7.12	0.092	121.71	6.72	128.48	5.74	6.77	5.99	**0.000**	0.563	0.172	**0.019**
NSL-Ms1mc (mm)		58.89	2.43	60.25	2.89	1.35	1.12	**0.000**	59.16	2.59	60.66	2.83	1.50	0.83	**0.000**	0.757	0.798	0.619
NSL-Ms2mc (mm)		47.40	2.75	49.05	2.83	1.64	1.17	**0.000**	48.11	3.64	50.01	3.97	1.90	0.90	**0.000**	0.581	0.251	0.251
OLp-Ms1 (mm)		49.00	2.42	48.21	3.14	−0.78	1.72	**0.030**	51.13	4.00	49.16	4.53	-1.98	1.71	**0.000**	0.089	0.492	0.066
OLp-Ms2 (mm)		40.48	2.28	39.78	2.84	−0.70	1.77	0.098	42.29	3.39	40.41	3.87	-1.87	1.61	**0.000**	0.147	0.563	0.079

SD, standard deviation.

P* = Wilcoxon test; *P* = Mann–Whitney *U*-test.

### Panoramic analysis

The results of the angular measurements in the panoramic analysis are presented in Table 4. During the therapy in the H group, the maxillary second molars were found to have tipped in the distal direction compared to the midline (8.30 degrees, SD 7.82 degrees, *P* = 0.001 and 6.36 degrees, SD 9.00 degrees, *P* = 0.008 on the right and left, respectively) and to the condylar line (8.29 degrees, SD 8.41 degrees, *P* = 0.001 and 6.37 degrees, SD 8.62 degrees, *P* = 0.003 on the right and left, respectively), with great individual variability. At the end of the study, significant differences were found between the L and H group. The angle between the midline and perpendicular line to the occlusal line of the right second maxillary molar (midline/RMs2p) was larger in the H group (24.79 degrees, SD 7.89 degrees and 31.09 degrees, SD 9.71 degrees, *P* = 0.037 in the L and H group, respectively). Alteration in angle midline/RMs2p (0.93 degrees, SD 7.33 degrees and 8.30 degrees, SD 7.82 degrees, *P* = 0.005 in the L and H group, respectively) and in angle condylar line/RMs2p (1.56 degrees, SD 7.10 degrees and 8.29 degrees, SD 8.41 degrees; *P* = 0.016 in L and H group, respectively) were more prominent in the H group compared to the L group, with great individual variability.

**Table 4.** Angular measurements in panoramic radiographs. In the H group, maxillary second molars tipped in the distal direction with great individual variability during HG therapy. The change during the therapy was larger in the H group

Panoramic analysis		Light force 300 g							Heavy force 500 g							L vs. H	L vs. H	L vs. H
		T1		T2		T1-T2		P*	T1		T2		T1–T2		P*	*P*	*P*	*P*
*N* = 40	Valid	22		22		22		22	18		18		18		18	40	40	40
		Mean	SD	Mean	SD	Mean	SD		Mean	SD	Mean	SD	Mean	SD		T1	T2	T1–T2
		Right side																
Midline/RMs2p (°)		23.86	8.19	24.79	7.89	0.93	7.33	0.924	22.79	8.54	31.09	9.71	8.30	7.82	**0.001**	0.619	**0.037**	**0.005**
Midline/RMs1p (°)		13.09	5.92	13.56	5.71	0.47	6.36	0.656	10.98	4.06	13.90	6.13	2.91	6.27	0.108	0.119	0.989	0.140
Condylar line/RMs1p (°)		103.20	4.98	104.30	5.48	1.10	6.27	0.899	101.35	4.58	104.29	6.19	2.94	6.35	0.130	0.190	0.882	0.286
Condylar line/RMs2p (°)		113.97	7.69	115.53	7.81	1.56	7.10	0.566	113.19	8.94	121.48	10.03	8.29	8.41	**0.001**	0.527	0.058	**0.016**
		Left side																
Midline/LMs2p (°)		28.85	7.47	30.71	8.24	1.86	6.48	0.248	27.58	10.67	33.94	8.00	6.36	9.00	**0.008**	0.798	0.106	0.155
Midline/LMs1p (°)		14.07	4.97	16.89	4.73	2.82	6.11	0.079	13.43	6.63	16.41	3.97	2.99	6.96	0.181	1.000	0.697	0.989
Condylar line/LMs1p (°)		103.95	5.22	106.14	5.11	2.19	6.41	0.290	103.02	5.42	106.02	4.13	2.99	7.04	0.130	0.600	0.677	0.882
Condylar line/LMs2p (°)		118.74	7.55	119.97	8.28	1.23	6.65	0.483	117.18	10.12	123.54	7.25	6.37	8.62	**0.003**	0.697	0.089	0.075

SD, standard deviation.

P* = Wilcoxon test; *P* = Mann–Witney *U*-test.

#### Development stage of the maxillary second molar

According to the panoramic analysis (Table 5), the development stage of the second molars did not have an impact on the distal movement of the first molars [OLp–Ms1: correlation coefficient (cc) = −0.155; *P* = 0.341]. If the second molars were initially (T1) in a more advanced development stage, they moved less in a distal direction (distance OLp–Ms2: cc = −0.318; *P* = 0.046) but tilted more easily during therapy (midline/RMs2p: cc = 0.335; *P* = 0.035; midline/LMs2p: cc = 0.351; *P* = 0.026; condylar line/RMs2p: cc = 0.325; *P* = 0.041; condylar line/LMs2p: cc = 0.401; *P* = 0.010). Tilting was statistically significant in the H group.

**Table 5.** Spearman’s rank correlation coefficients of changes during the study period T1–T2 compared to the development and eruption stage of the permanent maxillary second molar at T1. The second molars development stage did not have an influence on the first molar movement, but in more advanced development stage they moved less and tilted more easily in a distal direction

	Development stage of maxillary second molar at T1			Eruption stage of maxillary second molar at T1		
	Light force (300 g)	Heavy force (500 g)	Total	Light force (300 g)	Heavy force (500 g)	Total
*N*	22	18	40	22	18	40
Panoramic analysis						
Right						
Angle midline/RMs2p	0.264	0.351	0.335*	0.050	−0.323	−0.225
Angle midline/RMs1p	−0.232	0.082	−0.061	0.349	−0.012	0.077
Angle condylar line/RMs1p	−0.223	0.124	−0.045	0.349	−0.036	0.083
Angle condylar line /RMs2p	0.286	0.295	0.325*	0.075	−0.275	−0.197
Left						
Angle midline/LMs2p	−0.027	0.742***	0.351*	0.149	−0.454	−0.192
Angle midline/LMs1p	−0.057	0.072	0.001	0.037	−0.102	−0.044
Angle condylar line/LMs1p	−0.073	−0.007	−0.043	0.000	−0.081	−0.040
Angle condylar line/LMs2p	0.062	0.783***	0.401**	0.167	−0.530*	−0.223
Cephalometric analysis						
Angle NSL/Ms1p	0.072	0.276	0.191	0.111	−0.248	−0.099
Distance NSL-Ms1mc	0.407	−0.620**	0.046	0.149	−0.362	0.256
Distance OLp-Ms1	−0.111	−0.138	−0.155	0.000	0.114	0.086
Angle NSL/Ms2p	−0.052	0.284	0.182	−0.167	−0.303	−0.270
Distance NSL-Ms2mc	0.534**	0.340	0.499***	0.401	−0.311	−0.098
Distance OLp-Ms2	−0.169	−0429	−0.318*	−0.111	0.295	0.152

*P < 0.05, **P < 0.01, ***P < 0.001.

In the H group, the maxillary first molars erupted less during therapy if the development stage of the second molars was more advanced (distance NSL–Ms1mc: cc = −0.620; *P* = 0.006). The second molars erupted more if they were more developed (distance NSL–Ms2mc: cc = 0.499; *P* = 0.001).

#### Eruption stage of the maxillary second molars

The eruption stage of the maxillary second molars at the start of the CHG therapy did not influence the position of the first and second molars during treatment (Table 5). Of note is that, at T1, the occlusal surface was of the second molar at most at same level as the cementoenamel junction of the adjacent first molar (Table 1).

## Discussion

This controlled clinical trial was conducted in order to establish how force magnitude in CHG treatment influences the position of the maxillary first and second molars. The children were in pre-puberty at the start of the study and were divided into two study groups with light (300 g) or heavy (500 g) force in the CHG. The long outer arm of the face bow was bent upwards by 10–20 degrees with the aim of preventing distal tipping of the maxillary first molars (42). After a 10-month CHG therapy period, the patients were re-evaluated for future treatment. In most cases, a Class I molar relationship was achieved, and treatment was continued using fixed orthodontic appliances.

In our previous study, adherence to instructions was found to be better with a light force in extra-oral traction than with a heavy force (32). The children used CHG with a light force significantly more than with a heavy force during the study period: over 9 hours and less than 8 hours in the L and H groups, respectively. In addition, the groups were found to be initially homogenous and the effectiveness of the similarity of CHG therapy was based on a cephalometric analysis (16). The same treatment outcome, including skeletal (restriction in the anterior displacement of the maxilla) and dental (distal movement of the maxillary first molars) effects, can be achieved with a light or heavy force in CHG. The treatment outcome was the same even when CHG was used less in the H group.

One aim of CHG treatment is to distalize the maxillary first and second molars to a Class I molar relationship. As in all orthodontic treatment, it is important to achieve a stable treatment outcome. Thus, it is important to find an optimal appliance activation in order to gain maximum benefit from the therapy and minimize any negative side effects.

### The effects of CHG activation on the movement of the first and second maxillary molars

Only a few studies have examined the influence of force magnitude on the position of the maxillary first and second molars in CHG therapy (20, 43). Based on the present cephalometric analysis, the distal movement of the maxillary first molars was the same with a light and a heavy force and second molars move with them, but with great individual variability. Distal movement does not seem be totally bodily movement, as also found in previous studies (4, 5, 42–44). Using a heavy force, the molars appear to tilt more easily in the distal direction than with a light force. Tipping of the second molars was also detected in the panoramic analysis but only in the H group. Using a heavy force, tipping of the molars was detected even though the device was used less hours during the study period. It is possible that the long outer arms of the facebow bent downwards in the event of a heavy force during use, thereby allowing the first molars to tip distally, despite the outer arms being bent upwards to prevent side effects (10, 43). Based on this study, the second molars appear to follow the angular changes of the first molars (44). During HG post-treatment period, straightening and mesial migration of the first and second molars is expected (4,5,11). During normal occlusal development, mesial migration of the molars has been noted as well (45).

A larger extrusion of the first molars was expected in the H group compared to the L group because of the greater change in angulation and more extrusive force component. However, no differences were found between the groups. In our previous study, no posterior rotation of the lower jaw or change in overbite were observed, indicating no side effects of CHG therapy in the vertical direction (16). Masticatory forces and anterior rotation of the mandible could have prevented extrusion of the first molars during therapy (11, 17, 46).

### Eruption stage of the second maxillary molar

Based on previous studies, distalization of the maxillary first molars in HG therapy is more effective when the second molars have not yet erupted compared to after their eruption into the oral cavity (7, 20–22). The effect of the eruption stage of the second maxillary molars could not be properly studied because of the small variability in the eruption stage of the second molars as stated in the Results.

### Development stage of the second maxillary molar

Based on our results, the development stage of the second maxillary molar did not have an impact on the distal movement of the first molar, although the development stage appears to affect the eruption of the second molars. At a more advanced development stage, less distalization but more eruption was detected in the second molars during the study period. Increased root length has been suggested to enhance resistance, complicating distalization (22). A more advanced development stage of the second molars can be associated with easier tipping of these teeth, particularly with a heavy CHG force.

Most of the distalizing force of the CHG is focussed on the first molars and less on the adjacent second molars, which, despite less force, also move in the distal direction (20). It is considered that, at the early stage of formation, the second molars cannot prevent tipping of the first molars using a heavy force (20). In our study, more tipping of the first molars with a heavy force was noted. However, the relationship between tipping and the early development stage of the second molars was not confirmed.

Eruption of the second molars can be delayed by distalization of the first molars (6, 18). Distally tilted first molars may temporarily block the eruption path of the second molars (6, 19). In the present study, the extrusion/eruption of the first and second molars was found in both groups, with no differences between the groups at the end of the study. During the 10-month follow-up, it was not possible to investigate whether distalization or tipping affected the eruption of the second molars into the oral cavity. At the end of the study, only a few patients had partially erupted second molars. Further investigation is necessary in order to study how different force magnitudes in early CHG therapy potentially influence the eruption of the second molars.

The present study has clinical relevance; according to the present findings, light force in CHG cause less harmful side effects than with heavy force. This study confirms our previous finding to recommend light force in CHG therapy (16,32).

## Limitations

The lack of a control group is a true limitation of the study. To follow at same time, the maxillary first and second molar change in position (angular, sagittal, and vertical) in a similar age group was not possible; the ethical committee would not accept a control group having lateral cephalograms and panoramic radiographs with 10 months interval.

It would have been propriate to evaluate the inclination of the first molars on a digital model before and after the study and to find out how tipping is detectable clinically on the first molars. Lack of a suitable software to measure inclination angle has prevented us so far to investigate the change on digital models.

## Conclusion

According to this controlled clinical CHG trial, it can be concluded that:

1. With a heavy force, the maxillary first and second molars appear to tilt more easily in a distal direction compared to a light force, even if the device was used less and the long outer bows were bent 10–20 degrees upwards.
2. Unerupted maxillary second molars move with the first molars and do not appear to affect the movements of the first molars.
3. At a more advanced development stage in the second molars, less distalization was noted but there was more eruption during the treatment period.
4. Light force is recommended to use in CHG therapy because of minor side effects during the treatment.

## Conflict of interest

None to declare.

## Data availability

The data underlying this article cannot be shared publicly due to the privacy of the individuals that participated in the study. The data will be shared on reasonable request to the corresponding author.

## Supplementary Material

cjab010_suppl_Supplementary_Table
