# Supplementary material for: The impact of force magnitude on the first and second maxillary molars in cervical headgear therapy
Source: Eur J Orthod. 2021 Apr 3;43(6):648–57. doi: 10.1093/ejo/cjab010 (PMC10084717; doi:10.1093/ejo/cjab010)
Supplement: cjab010_suppl_Supplementary_Table [file cjab010_suppl_supplementary_table.docx]

|  |  | Difference in measurements 1 and 2 at T1 in the panoramic analysis | | | | | | | |
| --- | --- | --- | --- | --- | --- | --- | --- | --- | --- |
|  |  | **T1 Right** | | | | **T1 Left** | | | |
|  |  | **Midline/RMs2p (**°) | **Midline/RMs1p (**°) | **Condylar line /RMs1p (**°) | **Condylar line /RMs2p (**°) | **Midline/LMs2p (**°) | **Midline/LMs1p (**°) | **Condylar line/LMs1p (**°) | **Condylar line/LMs2p (**°) |
|  |  |  |  |  |  |  |  |  |  |
| N | Valid | 40 | 40 | 40 | 40 | 40 | 40 | 40 | 40 |
|  | Missing | 4 | 4 | 4 | 4 | 4 | 4 | 4 | 4 |
| Mean value of difference (absolute value) | | 2.45 | 2.71 | 2.27 | 2.53 | 4.43 | 2.55 | 2.04 | 3.65 |
| SD |  | 2.10 | 1.77 | 1.51 | 2.35 | 2.93 | 2.06 | 1.66 | 2.86 |
| ICC | | 0.927 | 0.826 | 0.851 | 0.914 | 0.837 | 0.85 | 0.903 | 0.873 |

ICC (absolute agreement, single measurements) was used to evaluate reliability of the first and second measurements.

|  |  | Difference in measurements 1 and 2 at T2 in the panoramic analysis | | | | | | | |
| --- | --- | --- | --- | --- | --- | --- | --- | --- | --- |
|  |  | **T2 Right** | | | | **T2 Left** | | | |
|  |  | **Midline/RMs2p (**°) | **Midline/RMs1p (**°) | **Condylar line/RMs1p (**°) | **Condylylar line/RMs2p (**°) | **Midline/LMs2p (**°) | **Midline/LMs1p (**°) | **Condylar line /LMs1p (**°) | **Condylylar line/LMs2p (**°) |
|  |  |  |  |  |  |  |  |  |  |
| N | Valid | 40 | 40 | 40 | 40 | 40 | 40 | 40 | 40 |
|  | Missing | 4 | 4 | 4 | 4 | 4 | 4 | 4 | 4 |
| Mean value of difference (absolute value) | | 2.66 | 2.49 | 2.34 | 2.30 | 3.65 | 2.86 | 2.33 | 3.12 |
| SD |  | 1.83 | 2.36 | 1.75 | 1.63 | 2.55 | 2.04 | 1.62 | 2.54 |
| ICC | | 0.942 | 0.846 | 0.879 | **0.959** | 0.865 | **0.717** | 0.83 | 0.886 |

ICC (absolute agreement, single measurements) was used to evaluate reliability of the first and second measurements.

|  |  | Difference in measurements 1 and 2 in the cephalometric analysis | | | | | | | | | | | |
| --- | --- | --- | --- | --- | --- | --- | --- | --- | --- | --- | --- | --- | --- |
|  |  | **T1** | | | | | | **T2** | | | | | |
|  |  | **NSL/Ms1p (**°) | **NSL-Ms1mc**  **(mm)** | **OLp-Ms1 (mm)** | **NSL/Ms2p (**°) | **NSL-Ms2mc (mm)** | **OLp-Ms2 (mm)** | **NSL/Ms1p (**°) | **NSL-Ms1mc (mm)** | **OLp-Ms1 (mm)** | **NSL/Ms2p (**°) | **NSL-Ms2mc (mm)** | **OLp-Ms2 (mm)** |
|  |  |  |  |  |  |  |  |  |  |  |  |  |  |
| N | Valid | 40 | 40 | 40 | 40 | 40 | 40 | 40 | 40 | 40 | 40 | 40 | 40 |
|  | Missing | 4 | 4 | 4 | 4 | 4 | 4 | 4 | 4 | 4 | 4 | 4 | 4 |
| Mean value of difference (absolute value) | | 3.21 | 0.67 | 1.01 | 3.59 | 0.70 | 0.83 | 3.15 | 0.72 | 1.46 | 4.16 | 0.63 | 1.197 |
| SD |  | 2.50 | 0.53 | 0.70 | 3.01 | 0.51 | 0.79 | 2.40 | 0.53 | 1.50 | 3.54 | 0.67 | 1.37 |
| ICC | | 0.769 | 0.943 | 0.936 | 0.743 | **0.964** | 0.928 | 0.769 | 0.952 | 0.864 | **0.692** | **0.964** | 0.864 |

ICC (absolute agreement, single measurements) was used to evaluate reliability of the first and second measurements.

Table. Mean values of difference in measurements 1 and 2 is presented in the table. The lowest ICC value was 0.692 (angle NSL/Ms2p) indicating at least moderate repeatability at measurements.
